# Ebola Virus Disease Outbreak — West Africa, September 2014

**Published:** 2014-10-03

**Authors:** Incident Management System Ebola Epidemiology Team, CDC; Ministries of Health of Guinea, Sierra Leone, Liberia, Nigeria, and Senegal; Viral Special Pathogens Branch, National Center for Emerging and Zoonotic Infectious Diseases, CDC

CDC is assisting ministries of health and working with other organizations to control and end the ongoing outbreak of Ebola virus disease (Ebola) in West Africa (1). The updated data in this report were compiled from ministry of health situation reports and World Health Organization (WHO) sources. Total case counts include all suspected, probable, and confirmed cases as defined by each country (2). These data reflect reported cases, which make up an unknown proportion of all actual cases. The data also reflect reporting delays that might vary from country to country.

According to the latest WHO update (2), a total of 6,574 Ebola cases had been reported as of September 23 from five West Africa countries (Guinea, Liberia, Nigeria, Senegal, and Sierra Leone) (Figure 1). The highest reported case counts were from Liberia (3,458 cases), Sierra Leone (2,021), and Guinea (1,074).

Geographic distribution of the number of Ebola cases reported during August 31–September 23 indicates that recent case counts continue to be high in the areas where Liberia, Sierra Leone, and Guinea meet (Figure 2).

Geographic distribution of the cumulative incidence of Ebola, as of September 23, indicates that the highest cumulative incidence (>100 cases per 100,000 population) was found in five districts in Guinea (Boffa, Dubreka, Gueckedou, Macenta, and Telimele), two districts in Liberia (Loffa and Margibi), and two districts in Sierra Leone (Kailahun and Kenema) (Figure 3).

The latest updates on the 2014 Ebola outbreak in West Africa, including case counts, are available at http://www.cdc.gov/vhf/ebola/outbreaks/guinea/index.html. The most up-to-date clinical guidelines on the 2014 Ebola outbreak in West Africa are available at http://www.cdc.gov/vhf/ebola/hcp/index.html.

## Figures and Tables

**FIGURE 1:**
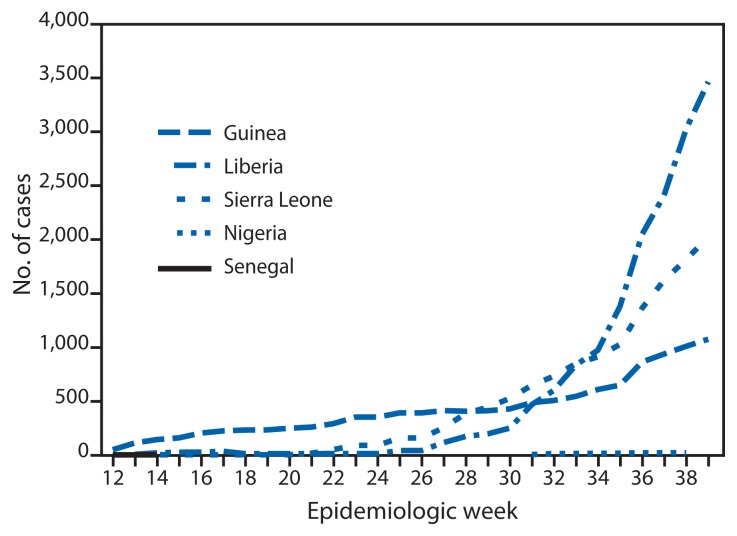
Cumulative number of Ebola virus disease cases reported — five countries, West Africa, March 29–September 20, 2014 **Sources:** Situation reports received from the ministries of health of Guinea, Liberia, Nigeria, Senegal, and Sierra Leone, and the World Health Organization.

**FIGURE 2:**
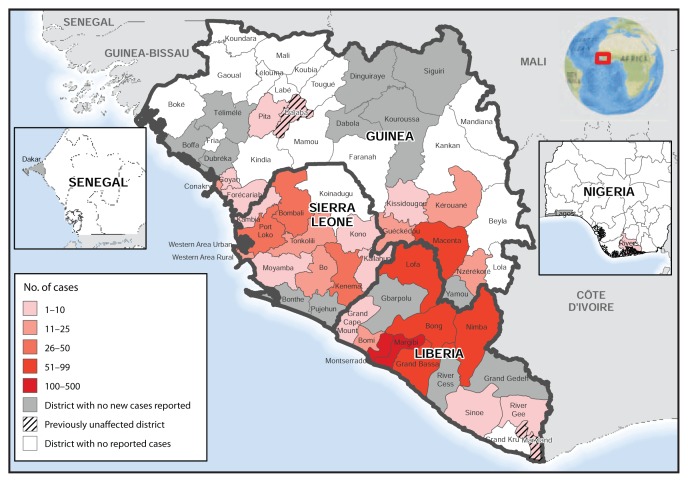
Number of new cases of Ebola virus disease reported — West Africa, August 31–September 20, 2014 **Sources:** Situation reports received from the ministries of health of Guinea, Liberia, Nigeria, Senegal, and Sierra Leone, and the World Health Organization.

**FIGURE 3:**
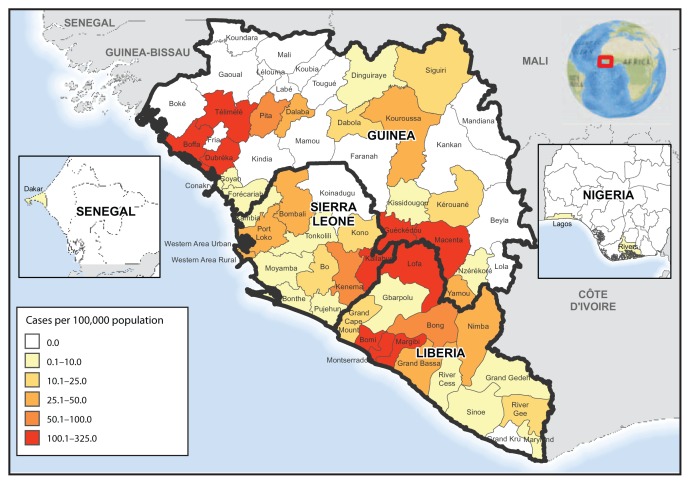
Ebola virus disease cumulative incidence* — West Africa, September 20, 2014 * Cumulative number of reported Ebola virus disease cases per 100,000 persons since December 22, 2013.
