# Errata

**Published:** 2014-10-10

## Vol. 63, No. 39

In the report, “Ebola Virus Disease Outbreak — West Africa, September 2014,” errors occurred. In Figure 1, the only Ebola case in Senegal was shown as having been reported during epidemiologic week 12. That case should have been shown as reported during epidemiologic **week 36**. In Figure 2, the title should read, “Number of new cases of Ebola virus disease reported — West Africa, **September 7**–20, 2014,” and the fifth entry in the legend should read, “100–**525**.”

